# Multiregional integration in the brain during resting-state fMRI activity

**DOI:** 10.1371/journal.pcbi.1005410

**Published:** 2017-03-01

**Authors:** Etay Hay, Petra Ritter, Nancy J. Lobaugh, Anthony R. McIntosh

**Affiliations:** 1 Rotman Research Institute, Baycrest Centre, Toronto, Ontario, Canada; 2 Department of Neurology, Charité–University Medicine, Berlin, Germany; 3 Bernstein Focus State Dependencies of Learning & Bernstein Center for Computational Neuroscience, Berlin, Germany; 4 Berlin School of Mind and Brain & Mind and Brain Institute, Humboldt University, Berlin, Germany; 5 MRI Unit, Research Imaging Centre, Centre for Addiction and Mental Health, Toronto, Ontario, Canada; 6 Division of Neurology, Department of Medicine, University of Toronto, Toronto, Ontario, Canada; 7 Department of Psychology, University of Toronto, Toronto, Ontario, Canada; Oxford University, UNITED KINGDOM

## Abstract

Data-driven models of functional magnetic resonance imaging (fMRI) activity can elucidate dependencies that involve the combination of multiple brain regions. Activity in some regions during resting-state fMRI can be predicted with high accuracy from the activities of other regions. However, it remains unclear in which regions activity depends on unique integration of multiple predictor regions. To address this question, sparse (parsimonious) models could serve to better determine key interregional dependencies by reducing false positives. We used resting-state fMRI data from 46 subjects, and for each region of interest (ROI) per subject we performed whole-brain recursive feature elimination (RFE) to select the minimal set of ROIs that best predicted activity in the modeled ROI. We quantified the dependence of activity on multiple predictor ROIs, by measuring the gain in prediction accuracy of models that incorporated multiple predictor ROIs compared to models that used a single predictor ROI. We identified regions that showed considerable evidence of multiregional integration and determined the key regions that contributed to their observed activity. Our models reveal fronto-parietal integration networks, little integration in primary sensory regions, as well as redundancy between some regions. Our study demonstrates the utility of whole-brain RFE to generate data-driven models with minimal sets of ROIs that predict activity with high accuracy. By determining the extent to which activity in each ROI depended on integration of signals from multiple ROIs, we find cortical integration networks during resting-state activity.

## Author summary

Models of fMRI activity can elucidate underlying dependencies that involve the combination of multiple brain regions. However, it remains unclear in which regions activity depends on unique integration of multiple predictor regions. To address this question, sparse (parsimonious) models could serve to better determine key interregional dependencies by reducing false positives. We used resting-state fMRI data, and for each brain region we performed whole-brain recursive feature elimination to select the minimal set of regions that best predicted activity in the region. We identified integrator regions by quantifying the gain in prediction accuracy of models that incorporated multiple predictor regions compared to single predictor region. Our study provides data-driven models that use minimal sets of regions to predict activity with high accuracy. By determining the extent to which activity in each region depended on integration of signals from multiple sources, we find cortical integration networks during resting-state activity.

## Introduction

Data-driven models constrained with functional magnetic resonance imaging (fMRI) data can elucidate some of the dependencies that involve a combination of multiple brain regions underlying the observed activity. Previous studies show that the activity in some brain regions during resting-state fMRI can be predicted with high accuracy using the activities in other regions [1]. The activity in each region of interest (ROI) can be modeled as a weighted sum of the activities of predictor ROIs (“features”), and the weights are found by optimization methods. Weights estimate the contribution of activity in predictor ROIs to activity in the modeled ROI, and can give clues about the connectivity between the regions (either indirect or direct) with respect to signal integration.

While previous models of ROI resting-state activity achieved high prediction accuracy [1], it remains unclear in which ROIs resting-state activity depends on multiregional integration. Some degree of prediction accuracy can be achieved using the predictor ROI most correlated with the modeled ROI. Therefore, it is important to identify ROIs whose activity exhibits unique integration of multiple varied signals (in contrast to activity that is redundant with other ROIs and/or integrates fewer varied signals). Estimating dependencies between ROIs is challenging due to the low temporal resolution and correlations between regions that can increase false positives. Previous studies used support vector regression to find ROI dependencies, in a manner that was inclusive in terms of predictors [1]. Finding a minimal set of ROIs that predict activity in a modeled ROI with high accuracy could serve to reduce false positives and thus better estimate the key ROIs involved and their contribution to the observed activity in the given ROI. To address this question, methods of feature selection, which generate sparser models and relate prediction accuracy to the number of predictors in the model, are particularly useful.

One commonly-used method is recursive feature elimination (RFE), a backward feature selection method that generates models iteratively, eliminating one or more predictors in each iteration–commonly the predictor with smallest weight, which least influences the model [2,3]. By examining the effect of elimination on validation error, RFE can be used to exclude redundant and spurious predictors, to arrive at a model with the minimal set of predictors that yields the most accurate prediction. RFE was originally used to classify cancer genes [3], and more recently was used successfully to classify brain states and conditions (e.g., resting vs. task or disease vs. control) from fMRI activity in predictor ROIs [4–9]. However, the method has not been used thus far to fit and predict activity in modeled ROIs from the activity in predictor ROIs.

Another method of feature selection, “least absolute shrinkage and selection operator”, or Lasso, uses L1 norm regularization of the regression. Regularization constrains properties of the predictor weights, and generally yields models with better prediction accuracy than ordinary least-squares regression [1,10,11]. L1 norm regularization minimizes the magnitude of the weights, and therefore yields sparse models with fewer redundant and spurious predictors [12,13]. As such, Lasso has been successfully applied to fMRI data for feature selection, disease prediction and classification [14–18], though it has not been used thus far to fit and predict activity in modeled ROIs from the activity in predictor ROIs. Whereas RFE examines directly the relationship between model prediction accuracy and the number of predictors in the model, Lasso relates prediction accuracy to the magnitude of predictor weights.

While feature selection methods such as RFE and Lasso are useful in addressing the challenging issue of spurious predictors (reduce false positives), other methods such as elastic net use additional L2 norm regularization to reduce missing true predictors (reduce false negatives), although at some cost to the reduction of false positives [11]. The choice of which modeling method to use depends on the question at hand. In this study we were interested in finding the predictors on which activity strongly depended (reduce false positives), and accordingly chose RFE and Lasso as the primary modeling methods, and used elastic net models to corroborate our results.

It is noteworthy that while the covariance matrix or partial correlations provide information about potential dependencies between pairs of ROIs [19,20], models derived from the activity data by ROI selection infer the number and combination of multiple ROIs that are jointly necessary for explaining/predicting the activity in a given ROI. This information cannot be derived just from looking at the correlations.

In this study, we first compared different methods for modeling ROI activity, in terms of prediction accuracy and feature selection. We used resting-state fMRI activity data from 46 subjects, and for each ROI per subject we generated models using whole-brain RFE, Lasso or elastic net to select the minimal set of ROIs that best predicted activity in the ROI. We then determined the degree to which ROI activity depended on multiple predictor ROIs, by comparing prediction accuracy of models incorporating multiple predictor ROIs to models that used a single predictor ROI. We identified ROIs that showed evidence of multiregional integration, and determined their key predictor ROIs, thereby identifying integration networks during resting state.

## Results

**Fig 1:**
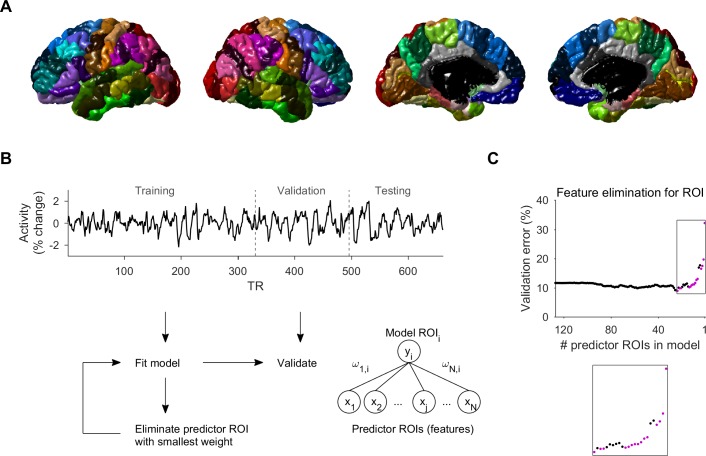
Recursive feature elimination for a region of interest. **A.** Brain volume was divided to 128 ROIs using anatomical features (see Methods). Lateral and medial views of both hemispheres show the 112 cortical ROIs. **B.** The activity data was divided into segments (training, validation and testing). The model for an ROI expressed the activity as a weighted sum of the observed activities in predictor ROIs (“features”), and predictor weights that best fitted the training data segment were found by regression. Models were generated iteratively, and in each iteration the predictor ROI with smallest weight was eliminated. The validation error of each model was assessed using the validation data segment. **C.** Validation error (in % of the activity variance) of each model for the ROI during the RFE process as a function of the number of predictor ROIs in the model. Models in magenta are from iterations where predictor elimination resulted in an increase of the smallest validation error across the rest of the iterations that followed. These predictor ROIs were used to generate the final model. In an alternative method (RFE2), the final model was the one with the minimum validation error during the recursive elimination process (minimum of the curve).

We used resting-state fMRI activity data of 46 subjects (~20 minutes in length per subject), and divided each brain volume to 128 ROIs (Fig 1A, see Methods). We first compared different methods for feature selection and modeling of ROIs (for each ROI per subject), in terms of model prediction error and the number of predictor ROIs selected. Two of the methods used RFE for feature selection, the third used Lasso, and the fourth used elastic net (see Methods). In the RFE approach (Fig 1B and 1C), the validation error of models generally decreased as predictor ROIs were removed, reaching a minimum beyond which elimination of predictors generally increased the validation error. One method to generate the final model for the ROI (which we termed RFE2), was to use the model that had the minimal validation error during the elimination process (Fig 1C, the minimum of the curve). However, due to fluctuations in the validation error curve, even after the minimum there were decreases in validation error that followed some of the increases, indicating that not all the predictor ROIs of the model at the minimum were necessary. Therefore, in the primary RFE method that we used, the final model for the ROI was generated using only predictor ROIs whose elimination increased the smallest validation error across the rest of the iterations that followed (Fig 1C, magenta dots).

**Fig 2:**
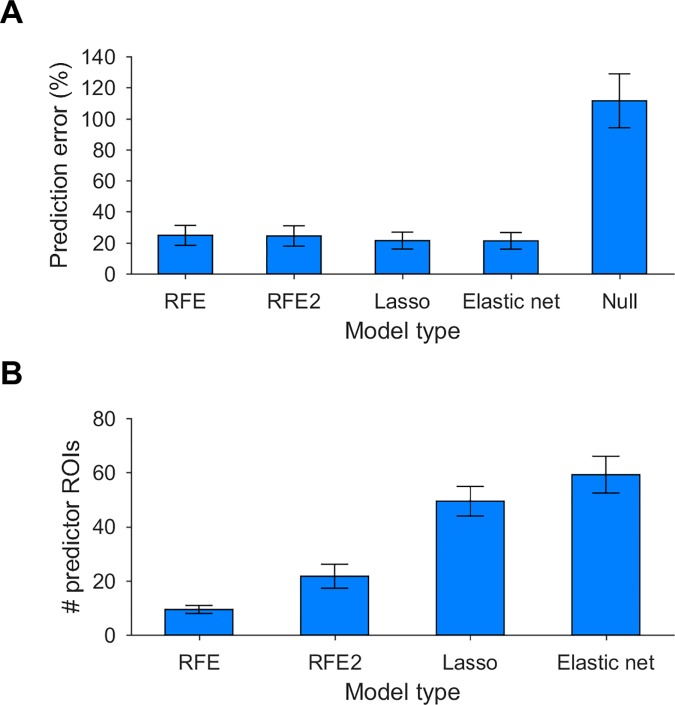
RFE models perform similarly to Lasso and elastic net models, but with fewer predictor ROIs. **A.** The average prediction error (across ROIs and n = 46 subjects) of models generated using RFE, RFE2, Lasso or elastic net was similar (average difference < 3% of activity variance), and significantly smaller than null models (average difference ~ 90%). Statistics reflect 108/112 cortical ROIs (excluding temporal pole and entorhinal cortex that had large prediction error in all models). **B.** The average number of predictor ROIs selected by RFE models was significantly smaller than that selected by RFE2, Lasso or elastic net models.

After generating models for ROIs using the different methods mentioned above, we assessed their prediction error using the test data segment, which was not used in any stage of model generation. The average prediction error across 108/112 cortical ROIs (excluding temporal pole and entorhinal cortex that had particularly large errors in all models, see below) was similar for the different modeling methods (24.8 ± 6.4% of the activity variance for ROIs modeled using RFE, 24.4 ± 6.5% for RFE2, 21.4 ± 5.4% for Lasso, and 21.2 ± 5.4% for elastic net, Fig 2A). Prediction error of null models (see Methods) was significantly larger (111.5 ± 17.4%, chance level = 100%, *p* < 10^−79^). For all ROIs, Lasso and elastic net models had comparable prediction error (difference < 1%), which was also slightly smaller than the prediction error of RFE models. However, for most (95/108) ROIs the difference between RFE and Lasso/elastic net ranged between 0.1–5.3%, and was therefore negligible in terms of effect size. The 13 ROIs with slightly larger difference (5–8%) had large prediction error in Lasso/elastic net models (25–36%), and were therefore not well-modeled by any of the methods. Thus, performance of models generated by the different methods was similar.

In contrast to the similarity in prediction error between the different modeling methods, RFE selected considerably fewer predictor ROIs (9 ± 1) compared to RFE2 (22 ± 4, *p* < 10^−64^), Lasso (49 ± 5, *p* < 10^−104^) or elastic net (59 ± 7, *p* < 10^−101^, Fig 2B). Thus, RFE yielded models with similar prediction accuracy as the other methods, and had fewer redundant predictor ROIs. In light of our aim at reducing false positives to better elucidate key multiregional dependencies, we conducted the subsequent analyses using the parsimonious models derived with RFE. We also note that RFE had shorter runtime compared to Lasso and elastic net. Fitting models for each subject took ~10 minutes with RFE vs. ~1 hour with Lasso/elastic net (and for the entire dataset the difference in runtime was ~8 hours vs. ~50 hours, respectively). Models generated using simple regression had larger prediction error than models generated using the other methods (30.1 ± 8.2%), a significant difference both in terms of effect size and statistics (overall average difference of 6–9%, *p* < 10^−39^). Furthermore, simple regression models for all ROIs included the entire set of predictor ROIs and thus provided no selection of predictors. There was no correlation between prediction error of ROIs and their connectivity in terms of number of predictors (*r* = -0.03, *p* = 0.75).

**Fig 3:**
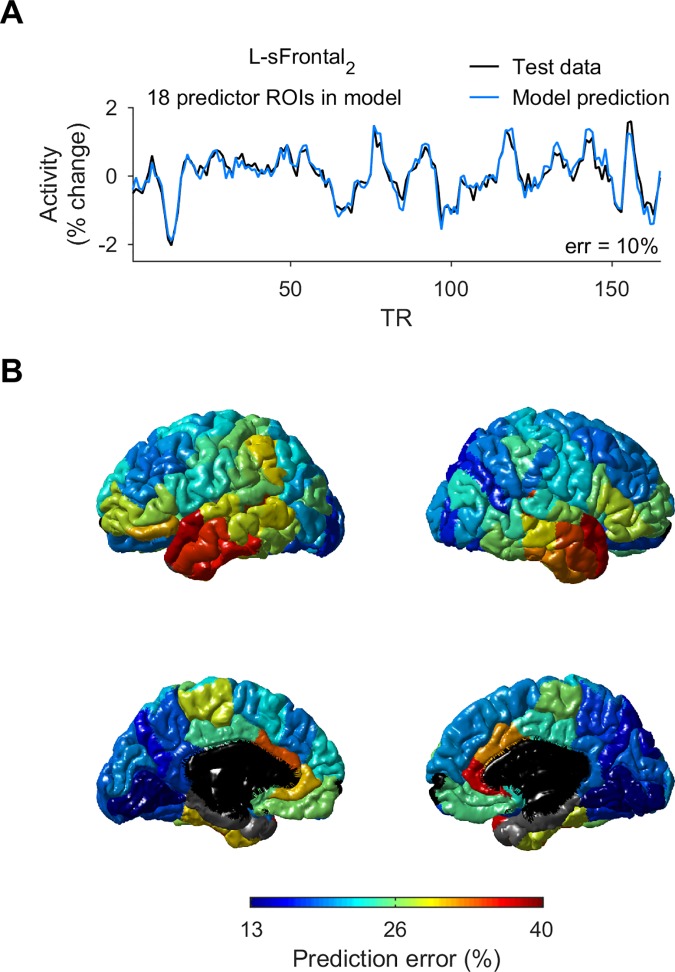
Model prediction error across ROIs. **A.** Observed activity (black) of left superior frontal gyrus_2_ (the second-most anterior ROI in left superior frontal gyrus, see Methods) of an example subject, and predicted activity of a model using 18 predictor ROIs selected by RFE (blue). **B.** Lateral and medial views of both hemispheres, showing the average prediction error in cortical ROIs (n = 46 subjects).

An example of model ROI with high prediction accuracy is shown in Fig 3A. Among models of the 112 cortical ROIs, small prediction errors (13–23%) were observed in 49 ROIs–in occipital cortex, superior and inferior parietal cortex, precuneus and cuneus cortex, isthmus and anterior cingulate, lingual gyrus, superior and rostral/caudal middle frontal gyrus (Fig 3B). Larger prediction errors (30–40%) were seen in models of anterior ROIs in temporal lobe (superior temporal gyrus_1_, middle temporal gyrus_1_, inferior temporal gyrus_1_), banks of superior temporal sulcus, and caudal/rostral anterior cingulate cortex. Otherwise, particularly large prediction errors (> 40%) were observed only for 4 ROIs (in each hemisphere)–temporal pole, parahippocampal gyrus, entorhinal cortex and transverse temporal cortex. Among subcortical ROIs, models of thalamus and cerebellum had small prediction errors (16–22%), whereas models of the pallidum, amygdala, and accumbens had large errors (> 40%). Prediction error was weakly inversely-correlated with the temporal signal to noise ratio (tSNR, see Methods) of the ROI (*r* = -0.33, *p* < 0.001, n = 128 ROIs). Importantly, the ROIs with particularly large prediction errors, such as temporal pole and entorhinal cortex, also had a small tSNR (0.92 and 0.71, respectively, where the tSNR across ROIs ranged from 0.71 to 1.99). In contrast, the anterior temporal and cingulate ROIs with prediction error between 30–40% mentioned above had tSNR of average magnitude (~1.3), except for the inferior temporal gyrus_1_, suggesting that resting-state activity in these ROIs depended on other regions in a more variable manner compared to other ROIs.

**Fig 4:**
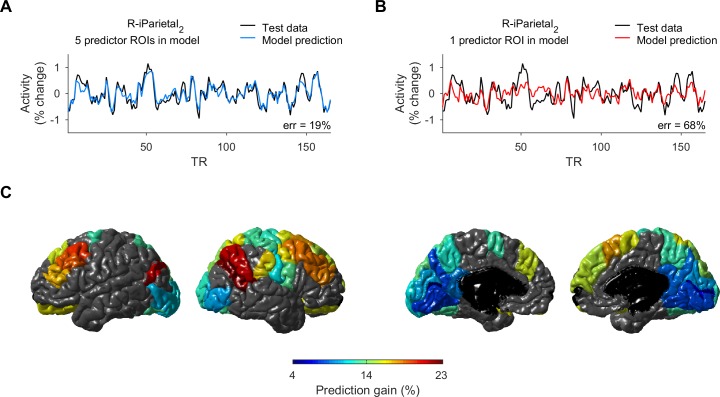
Multiregional prediction gain across ROIs. **A.** Observed activity (black) of right inferior parietal cortex_2_ (the middle ROI of inferior parietal cortex, see Methods) in an example subject, and predicted activity of a model using 5 predictor ROIs selected by RFE (blue). **B.** Observed (black) and predicted (red) activity for the same ROI, but with a model using 1 predictor ROI, the one most correlated with the ROI in the training data. The difference in prediction error of the two models for the ROI (the “multiregional prediction gain”) was 49% (*p* < 10^−15^). **C.** Lateral and medial views of both hemispheres, showing the average prediction gain in the 49 cortical ROIs that were well-modeled (prediction error 13–23% as in Fig 3). Other ROIs with larger prediction error are colored in gray.

To examine the dependence of activity on multiple predictor ROIs, we compared modeled ROIs in terms of a novel measure that we termed “multiregional prediction gain”–the improvement in prediction accuracy of a model that used multiple predictor ROIs compared to a model generated using the single predictor ROI most correlated with the modeled ROI (see Methods). For example, the model for activity in right inferior parietal cortex_2_ of an example subject shown in Fig 4A had prediction error of 19% using 5 predictor ROIs selected by RFE. An alternative model for the same ROI, generated using only the single predictor ROI most correlated with the modeled ROI in the training data, had a larger prediction error of 68% (Fig 4B). The multiregional prediction gain for this ROI was therefore 49% (*p* < 10^−15^, determined by t-test between the square errors of the two models), and thus there was evidence in the data of considerable dependence of ROI activity on the activities in multiple regions.

Among the 49 cortical ROIs that were well-modeled (average prediction error 13–23%), 20 ROIs, primarily parietal and frontal, had large prediction gain (13–23%, Fig 4C). These integrator ROIs included right superior frontal gyrus_1-4_, left superior frontal gyrus_2_, left/right rostral-middle frontal gyrus_2,3_, left/right caudal-middle frontal gyrus, left inferior parietal cortex_2_, right inferior parietal cortex_1,2_, right superior parietal cortex_2_, right supramarginal gyrus_2,3_, right precentral gyrus_2_, and right/left lateral orbital cortex. The large multiregional prediction gain in these ROIs reflected a significant difference between the prediction errors of models that had multiple predictor ROIs and the prediction errors of models that used only a single predictor ROI (*p* < 10^−6^), as determined by t-test corrected for multiple comparisons (see Methods). ROIs with moderate multiregional prediction gain (8%– 13%) included left/right precuneus cortex_2_, left/right fusiform gyrus_2_, left cuneus cortex, and left/right lateral occipital cortex_1-3_.

ROIs with small multiregional prediction gain (< 8%), and thus no dependence of activity on unique multiregional integration, included pericalcarine cortex, isthmus cingulate cortex, precuneus cortex_1_, and lingual gyrus. For these ROIs, the activity was strongly correlated with a single predictor ROI, indicating a strong coupling between the two ROIs, which masked evidence of dependence of the activity on other ROIs. For example, the small gain of left isthmus cingulate cortex was due to strong coupling with right isthmus cingulate cortex in 85% of the cases (with average activity correlation of 0.84 between the two ROIs), left precuneus cortex_1_ in 10% of the cases (activity correlation = 0.8), and left precuneus cortex_2_ in the rest of the cases (activity correlation = 0.77). For these ROIs, the data therefore indicated redundancy in terms of signal integration during resting state.

We note that whereas ROIs whose activity is highly correlated with other ROIs would have small prediction gain, ROIs that are well-modeled and whose activity depends on multiregional integration (large prediction gain) cannot be found just by looking at the activity correlation matrix. This is because ROIs that are only moderately correlated with other ROIs could differ in terms of how well their activity can be predicted from multiple regions, and therefore differ in their multiregional prediction gain. For example, the maximal activity correlation of right inferior parietal cortex_2_ with other ROIs was 0.78 and its average activity correlation was 0.42, its average prediction error was 17%, and its average prediction gain was 23.5%. In contrast, whereas the maximal and average activity correlations of left supramarginal gyrus_3_ with other ROIs were 0.78 and 0.42 as well, its average prediction error was higher (27%) and its prediction gain was lower (12%). Thus, the level of activity correlation did not in itself indicate the extent of multiregional integration or prediction error.

We also note that the magnitude of multiregional prediction gain need not necessarily be correlated with the number of predictor ROIs. For example, a multiregional prediction gain of 20% could theoretically depend on 2 predictor ROIs just as it could depend on 20 predictor ROIs. In the first case, each predictor ROI would be responsible on average for a larger portion of the prediction gain compared to the latter case (10% vs. 1%, respectively). Thus, a difference in prediction gain between ROIs could either be related or not to the number of predictor ROIs. We therefore examined the relationship between the multiregional prediction gain and the number of predictor ROIs in the models, and found that ROIs with larger multiregional prediction gain tended to include a larger number of predictor ROIs (*r* = 0.78, *p* < 10^−26^). Therefore, the different gain between ROIs corresponded to a difference in the number of predictor ROIs on which the activity depended, rather than a difference in the average contribution of predictor ROIs to the prediction gain. Multiregional prediction gain for an ROI was not correlated with the tSNR of its time series (*r* = 0.16, *p* > 0.05, n = 128 ROIs). We found no correlation between multiregional prediction gain and subject age in any of the ROIs (*p* > 0.05).

**Fig 5:**
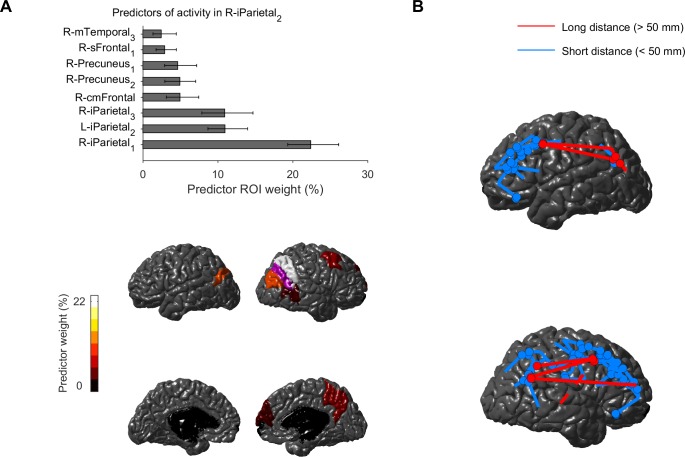
Key predictor ROIs reveal integration networks during resting-state activity. **A.** The weight of key predictor ROIs for models of right inferior parietal cortex_2_ (bootstrapping estimated mean and 95% confidence intervals). Bottom–lateral and medial views of left/right hemispheres, showing right inferior parietal cortex_2_ (magenta) and the weight of its key predictor ROIs. **B.** Integrator ROIs (with large multiregional prediction gain) and their key predictor ROIs formed interconnected integration networks. Short-distance dependencies (distance < 50 mm) are shown in blue, and long-distance dependencies (> 50 mm) are shown in red. Dashed lines are inter-hemispheric. Homotopic ROIs were often among the key predictors of integrator ROIs but their dependencies are not shown in this figure.

For each of the 20 integrator ROIs (with large multiregional prediction gain) mentioned above, we looked for key predictor ROIs on which the activity depended most (see Methods). In the analysis of predictor ROI weights, the weights were normalized as % of the total weight onto the modeled ROI (see Methods). For example, in models of right inferior parietal cortex_2_, key predictor ROIs included both neighbouring ROIs (right inferior parietal cortex_1,3_, right precuneus cortex_1,2_, right middle temporal gyrus_3_) and more distal ROIs (> 50 mm apart, right caudal-middle frontal gyrus, right superior frontal gyrus_1_), as well as the homotopic left inferior parietal cortex_2_ (Fig 5A). The set of integrator ROIs and their key predictor ROIs formed interconnected integration networks (Fig 5B) that involved primarily parietal and frontal ROIs, with long-distance (> 50 mm) connections between inferior parietal and caudal-middle/superior frontal ROIs. Key predictor ROIs were primarily ipsilateral to the integrator ROIs, and included also the homotopic ROI, but did not include subcortical ROIs. The weights of predictor ROIs were moderately correlated with the activity correlations between the predictors and modeled ROI (*r* = 0.76 ± 0.05, *p* > 10^−53^, determined by shuffling the activity correlations), further supporting the meaningfulness of the estimated weight. We did not find any correlation between subject age and predictor weights (*p* > 0.05 for all ROIs).

**Fig 6:**
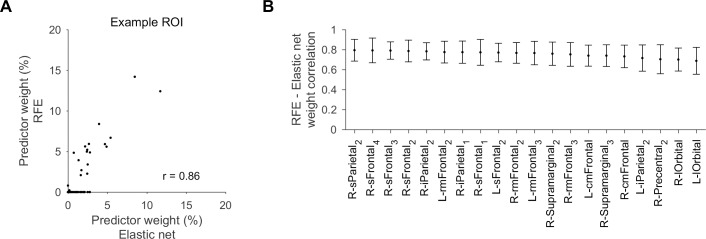
Predictor weights in RFE and elastic net models are strongly correlated. **A.** Predictor ROI weights for models of right inferior parietal cortex_2_ of an example subject, derived using either RFE or elastic net, were strongly correlated (*r* = 0.86, *p* < 10^−38^). **B.** Correlation between predictor ROI weights derived using RFE or elastic net for the 20 ROIs with large multiregional prediction gain.

To corroborate our results with modeling methods that do not prune predictors as heavily as RFE or even Lasso, we compared predictor ROI weights as determined by RFE to weights determined by elastic net. Predictor ROI weights were strongly correlated between the two types of models, with the difference mainly in that RFE pruned predictors that had small weights in models derived by elastic net (Fig 6A). For the 20 integrator ROIs highlighted above, the correlations between model weights derived using the two methods were 0.77 ± 0.03 on average, and ranged from 0.69 ± 0.13 to 0.79 ± 0.11 (Fig 6B). Thus, key predictor ROIs were identified to a similar degree by the two methods.

**Fig 7:**
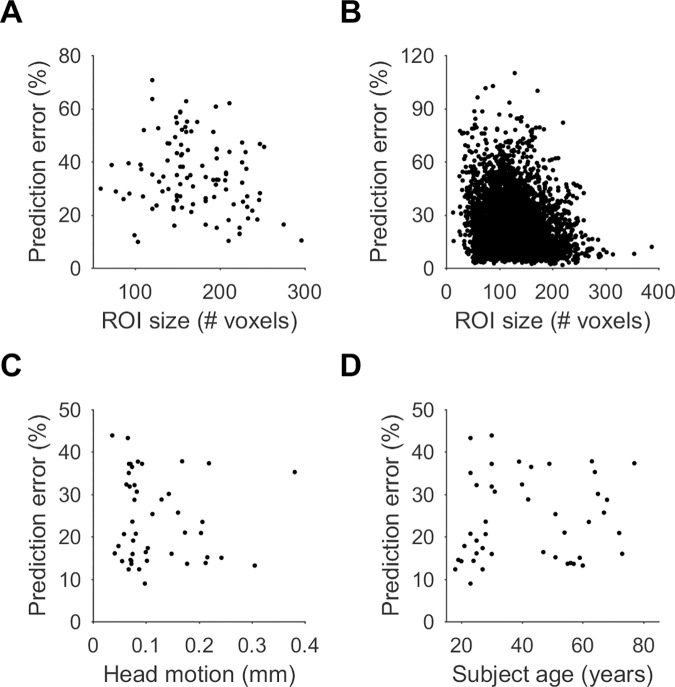
Prediction error did not depend on ROI size, head motion or subject age. **A, B.** Prediction error of ROIs was only weakly correlated with the ROI size, both in single subjects (**A**, *r* = -0.19, n = 104 cortical ROIs) and across subjects (**B**, *r* = -0.2, n = 4,784 cortical ROIs). **C.** Average prediction error was not correlated with subject head motion (*r* = -0.09, *p* = 0.56). **D.** Average prediction error was not correlated with subject age (*r* = -0.1, *p* = 0.49).

Prediction error of models for cortical ROIs (excluding the 4 ROIs in each hemisphere with large prediction error mentioned above) was only weakly correlated with ROI size, either for single subjects (*r* = -0.19, Fig 7A) or across subjects (*r* = -0.2, Fig 7B). Some correlation is to be expected by the de-noising effect of averaging more voxels in larger ROIs compared to smaller ROIs. While the estimated head motion was small (0.12 ± 0.07 mm, see Methods) but correlated with subject age (*r* = 0.71, *p* < 10^−7^), as is commonly the case [21], the average prediction error was not correlated with subject head motion (*r* = -0.09, *p* = 0.56, Fig 7C) or subject age (*r* = 0.1, *p* = 0.49, Fig 7D).

## Discussion

We used whole-brain recursive feature elimination to select minimal sets of ROIs that best predicted the resting-state fMRI activity in ROIs of each subject. We used these data-driven models to highlight ROIs whose activity depended considerably on multiple predictor ROIs, reflected in a substantial gain in prediction accuracy of models that used multiple predictor ROIs compared to single predictor ROI. We determined the key predictor ROIs that contributed to the activity in each integrator ROI, and showed that the integrator and predictor regions formed interconnected integration networks during resting state.

Multiregional prediction gain measures both the variety of signals that a region integrates and the uniqueness of this integration compared to other regions. Thus, a region that is well-modeled and whose activity integrates different streams of information more uniquely compared to other regions (less correlated with other regions) will have a larger multiregional prediction gain. In contrast, a region that integrates similar streams of information or whose activity is coupled (strongly correlated) to the activity of other regions will have a small multiregional prediction gain, so far as is evident in the data of activity during the particular brain state tested. Importantly, whereas high activity correlations are associated with small multiregional prediction gain, data-driven models are necessary to find ROIs with large multiregional prediction gain, and to infer the number and combination of multiple predictor regions on which ROI activity depends.

ROIs whose resting-state activity depended on multiple predictor ROIs were seen within superior frontal gyrus, rostral-middle frontal gyrus, caudal middle frontal gyrus, inferior parietal cortex, and lateral orbital cortex in both hemispheres, as well as right superior parietal cortex, right supramarginal gyrus, and right precentral gyrus. The large dependence on multiregional integration that we found in the frontal/parietal regions agrees with their involvement in higher cognitive functions such as reflection during resting state [22], and supports previous indications based on correlations that these regions are functional hubs during resting state [23,24]. The high prediction accuracy in these regions agrees also with previous modeling studies [1] and with their high activity during resting-state [25]. The integrator ROIs and their key predictor ROIs formed interconnected resting-state integration networks, consisting mostly of ROIs in the parietal and frontal lobes, in line with previous findings [23]. Our models indicate long-distance dependencies connecting the frontal and parietal networks between parietal cortex and middle/superior frontal gyrus. The activity in the integrator ROIs did not depend significantly on subcortical ROIs, indicating that during resting-state they integrated primarily cortical activity.

For some ROIs, the models predicted activity with high accuracy but did not depend on multiple predictor ROIs, due to strong and stable correlation (coupling) between the modeled ROI and a single predictor ROI. The size of ROIs was sufficiently large (e.g. 55 ± 14 voxels for pericalcarine or 74 ± 21 for isthmus cingulate) to be spatially well-separated to reduce the effect of spatial correlations between nearby voxels. Thus, the small multiregional prediction gain in these regions indicates a degree of redundancy among some regions during resting state in terms of informative activity (as in the case of isthmus cingulate cortex), and/or that the regions do not integrate numerous and varied cortical signals (as in the case of primary sensory regions such as pericalcarine cortex). While it is not possible to distinguish between these two possibilities solely based on multiregional prediction gain, the small gain can be informative along with other pieces of information. E.g., as isthmus cingulate cortex was previously shown to be a hub during resting state [26], the small multiregional prediction gain in our study indicates that its signal integration during resting state is redundant with that of homotopic and precuneus ROIs.

We have shown that RFE models predict resting-state activity with similar accuracy as Lasso or elastic net models, but with considerably fewer predictor ROIs (on average less than 10). RFE is therefore a useful whole-brain method for generating parsimonious models that predict activity with high accuracy, and for discovering key predictor ROIs in high-dimensional brain activity data. Future studies that focus on minimizing false negatives rather than false positives can use our framework of analyzing multiregional prediction gain and predictor ROIs with other types of models such as elastic net.

Our method is somewhat related to other multivariate methods such as partial correlations [19,20] and partial least squares analysis [27], with the advantage that our modeling method grounds the estimated interregional dependencies with their joint prediction of ROI activity. Future studies should systematically compare our method to other common multivariate methods of fMRI analysis, for the purpose of properly ascertaining the manner in which these methods complement one another. In addition, data-driven RFE models of ROI activity could complement connectivity analysis of pairwise correlations, by pruning ROIs that are correlated with the modeled ROI but are not necessary for predicting its activity and therefore are likely to be spurious.

The high prediction accuracy of RFE models indicates stability of integration of signals during resting state, which agrees with the previously reported stability in activity correlations between structurally connected regions [28]. High prediction accuracy was associated with errors of ~15%– 25% of the activity variance, indicating that 75%– 85% of the activity in these regions was driven by stable interactions. The prediction error could result from measurement noise as well as from activity driven by variable interactions on top of a stable core. Given that prediction error was only weakly inversely-correlated with tSNR of the ROI time series, it is likely that the error was more influenced by the variability in interactions between regions. Thus, for ROIs such as anterior superior/middle temporal gyrus, and rostral/caudal anterior cingulate cortex, where the error was larger (30–40%) and tSNR was average, it is likely that the resting-state activity depended on other ROIs more variably. Considerably large prediction errors (50–60%) were seen only in temporal pole, parahippocampal gyrus, entorhinal cortex and transverse temporal cortex, which are prone to susceptibility artifacts [29] and had small tSNR in our study. We did not observe significant relationships between subject age and prediction accuracy or multiregional prediction gain, indicating that the portion of stable interactions between regions was similar across a wide age span.

While care must be taken in interpreting predictor weights [30], in our analysis of predictor ROI weights we have used normalized weights (% of total weight onto the modeled ROI) rather than raw coefficients, to allow a meaningful comparison of weights within and between models. Furthermore, there are several indications that the predictor weights we estimated signify their importance in influencing the modeled ROI activity. First, the models predicted new data with high accuracy, indicating that the estimated weights capture true dependencies underlying the observed activity. Second, the weights of predictor ROIs were strongly correlated with the activity correlations between the predictor ROIs and modeled ROI, further supporting their meaningfulness.

Similarly to previous modeling studies [1], we show that despite the low temporal resolution of fMRI and the correlations between regions, training data segments that are not too long (~300 points, or ~10 minutes of scanning) can be used to generate models with high prediction accuracy for most regions. Nevertheless, future models will benefit from utilizing longer datasets (e.g., the Human Connectome Project [31]), which we expect will better constrain the models and improve determining the key dependencies between regions. Such datasets could also be used to corroborate the findings of this study. Other validations could be made by comparing the robustness of models and results using different parcellations [32,33], or testing the methods using ground-truth artificial data generated by brain simulation tools [34].

Future studies should utilize our data-driven modeling and analysis method to data acquired during other brain states (e.g. during task), to determine the dependence of activity on multiple predictor ROIs and the key regions involved. The interconnected sets of integrator ROIs and their predictor ROIs could also be analyzed in terms of network theoretic measures [35–37], relationship with structural connectivity and activity correlations [23,38–41], and temporal dynamics [42–44].

## Methods

### Experimental data

We used resting-state fMRI blood-oxygen-level dependent (BOLD) data from 46 healthy subjects (18 males, 28 females) of ages between 18 and 77 (41 ± 18 years, median = 35), acquired at Berlin Center for Advanced Neuroimaging, Charité University Medicine Berlin [34,45]. Subjects were recruited as volunteers and gave written informed consent before the study, which was approved by the local ethics committee in accordance with the institutional guidelines at Charité Hospital, Berlin, and performed in compliance with the Code of Ethics of the World Medical Association (Declaration of Helsinki), the relevant laws and institutional guidelines. Briefly, subjects were lying relaxed in a supine position inside the MR scanner. They were instructed to relax, lie still, and minimize movement (especially head movement). The subject’s head was immobilized with removable cushions (Siemens equipment), and earplugs were used to prevent adverse effects due to scanner noise. Subjects were instructed to close their eyes but not to fall asleep. Functional MRI (BOLD-sensitive, T2*-weighted echo planar imaging, TR 1940 ms, TE 30 ms, FA 78°, 32 transversal slices (3 mm), voxel size 3 × 3 × 3 mm, FoV 192 mm, 64 matrix) was recorded using a 3 Tesla Siemens Tim Trio MR scanner simultaneously with 64 channels EEG [46]. Each scan spanned ~20 min (661 TR). Subjects also underwent anatomical T1-weighted scans (magnetization-prepared rapid gradient-echo, TR 1900 ms, TE 2.25 ms, 192 sagittal slices (1.0 mm), voxel size 1 × 1 × 1 mm, FA 9°, FoV 256 mm, 256 matrix) as well as T2-weighted scans (2D turbo spin echo, TR 2640 ms, TE1 11 ms, TE2 89 ms, 48 slices (3.0 mm), voxel size 0.9 × 0.9 × 3 mm, refocusing FA 150°, FoV 220 mm, 256 matrix).

### Preprocessing of the fMRI data

Preprocessing was performed using FEAT (fMRI Expert Analysis Tool) Version 6.0 from the FMRIB (Functional MRI of the Brain) Software Library (FSL FEAT, 2014). It included correction for head motion using MCFLIRT [47], fieldmap correction to reduce spatial distortion of EPI images [48], BET brain extraction to remove non-brain tissue [49], and high-pass filtering (cutoff at 100s) to adjust for baseline drift of the signal. Functional data was registered to the individual high-resolution anatomical T1 images using FreeSurfer. The BOLD signal was not smoothed nor corrected for slice-timing differences, in line with suggestions by previous studies [50].

### Brain parcellation

We divided the brain to 128 gray matter ROIs (64 in each hemisphere, Fig 1A), 112 cortical and 16 subcortical (cerebellum, thalamus, caudate, putamen, pallidum, hippocampus, amygdala, and accumbens). The parcellation was conducted in two stages: the brain was first divided to 84 ROIs (68 cortical and 16 subcortical) according to anatomical features detected in the anatomical MRI scan, using an automated method described previously [51]. Next, 26 large cortical ROIs (13 in each hemisphere) were subdivided into several contiguous ROIs of equal size along the region’s main axis: Superior frontal gyrus (4 ROIs, anterior-posterior axis), inferior parietal cortex (3, superior-inferior), lateral occipital cortex (3, posterior-anterior), middle temporal gyrus (3, anterior-posterior), precentral gyrus (3, superior-inferior), rostral-middle frontal gyrus (3, anterior-posterior), superior parietal cortex (3, posterior-anterior), superior temporal gyrus (3, anterior-posterior), supramarginal gyrus (3, posterior-anterior), inferior temporal cortex (2, anterior-posterior), fusiform gyrus (2, anterior-posterior), postcentral gyrus (2, superior-inferior), and precuneus cortex (2, posterior-anterior). Subdivided ROIs of the larger regions were referred to in the text as region_x_, where x is the number of the ROI along the region’s main axis. For example, left superior frontal gyrus_2_ was the second-most anterior ROI of left superior frontal gyrus. Subdividing the large regions resulted in more similar sizes across ROIs (124 ± 40 vs. 211 ± 161 voxels). The activity in each ROI was the average over its voxels, converted to percent signal change (from the average of the time-series for that ROI).

### Modeling and feature selection

For each subject we split the time-series into three segments (Fig 1B): training data (first half), validation data (third quarter), and test data (last quarter). The model for each ROI expressed the activity as a weighted sum of the activities in other ROIs (“features”), or:

$$y_{i}=\sum_{j\neq i}\omega_{j,i}x_{j}$$ (1)

Where *x*_*j*_ is the observed activity in predictor ROI_j_, and *ω*_*j*,*i*_ is the weight of predictor *x*_*j*_. We used RFE to select the minimal set of ROIs that best predicted the validation data (Fig 1). The feature selection algorithm involved the following steps:

1. Begin with the set of all predictor ROIs except the modeled ROI_i_, *S* = {*x*_*j*_}_*j* ≠ *i*_.
2. Generate model using set *S*, finding predictor weights *ω*_*j*,*i*_ by fitting the training data using regression (in Matlab) of the observed activity of ROI_i_ and the predictor ROIs in set *S*.
3. Eliminate the predictor ROI with smallest weight from the set *S*
4. Repeat steps 2–3 until only one predictor ROI remains

The prediction error (see below) of each model was assessed using the validation data segment, and referred to as the validation error. The algorithm yielded a set of models (each having one less predictor ROI than the previous) and their corresponding validation errors (Fig 1C). At this point we considered two methods to yield the final model for the ROI. In one method (which we termed RFE2), the final model was the model with smallest validation error during the elimination process (the minimum of the validation error curve, Fig 1C). Because the validation error fluctuated, with decreases in error following increases, we also considered another method (which we termed RFE) to further eliminate redundant predictor ROIs, whereby we selected only the predictor ROIs whose elimination resulted in increase of the smallest validation error across the rest of the iterations that followed, and we used these predictor ROIs to generate a new final model for the ROI, fitting the training data by regression.

We also generated for each ROI a third model using Lasso (in Matlab), a different feature selection method, to compare with RFE. Lasso adds a regularization term to the regression, the L1 norm of the feature weights, which minimizes the size of weights and reduces the number of predictors [12], and by avoiding extreme parameter values reduces overfitting and increases prediction accuracy [1,10,11]. Lasso minimizes:

$$\langle {\left(y_{i}-x_{i}\right)}^{2}\rangle +c|\omega |$$ (2)

where *y*_*i*_ is the model activity of ROI_i_ (Eq 1), *x*_*i*_ is the observed activity of ROI_i_, *c* is the magnitude of regularization, and |*ω*| is the L1 norm of the weight vector. For each ROI, we generated models using 200 different magnitudes of the regularization and consequently a different number of predictor ROIs in the models. We tested the candidate models on the validation data segment, and selected as the final model the one with smallest validation error.

We corroborated our results using a fourth modeling method, elastic net (in Matlab), that prunes predictors less strongly than RFE or Lasso and thus can reduce false negatives [11]. Elastic net uses both L1 and L2 norms of the predictor weights vector to regularize the regression, selecting groups of correlated predictors together instead of discarding redundant predictors. Elastic net therefore has an additional parameter compared to Lasso, namely the relative contribution of L1 vs. L2 norms (ranging from 0 to 1). To keep the runtime sensible, for each ROI we generated models similarly to Lasso, but searched 50 magnitudes of the regularization for each of 5 magnitudes of the L1 vs. L2 contribution (0.01, 0.05, 0.1, 0.5, and 1), or 250 different combinations overall.

### Prediction error

We measured prediction error in % of the activity variance, the mean-squares error between the observed and predicted time-series, normalized by the total mean squares of the observed time series, or:

$$\langle {\left(y_{i}-x_{i}\right)}^{2}\rangle /\langle {\left(x_{i}-\bar{x_{i}}\right)}^{2}\rangle$$ (3)

where *x*_*i*_ is the observed activity in the ROI_i_ and *y*_*i*_ is the model activity in the ROI_i_ (Eq 1). Prediction error of candidate models during model generation was determined using the validation data segment and referred to as validation error (see above), whereas prediction error of the final model was determined using the test data segment, which was not used during model generation. The significance of prediction error was assessed using null models, by calculating the prediction error when randomly reordering the weights of predictor ROIs in the model for the ROI (500 permutations per ROI).

### Multiregional prediction gain

For each ROI, we calculated the difference between the prediction error of its multiple-predictors model and the prediction error of a model generated using only the predictor ROI most correlated with the modeled ROI in the training data. We termed the difference as “multiregional prediction gain”, since it measured the improvement in prediction accuracy using the multiple-predictors model. The multiregional prediction gain was therefore a measure of the effect size for the difference in prediction errors of the two types of models, and was reported in this study together with its p-value significance which was determined using t-test on the square errors of the two models. Because effect size is an important factor in determining significance, we focused in this study on highlighting cases where the gain was both large and significant.

### Bootstrapping

To establish robust estimates of some measures across subjects (e.g., predictor ROI weights), we estimated the average across subjects using bootstrapping in Matlab, by sampling 500 times with replacement to estimate the measure’s mean and the 95% confidence intervals.

### Key predictor ROIs

For each modeled ROI, we determined key predictor ROIs that had significantly large weights, as established by t-test (see below) vs. weights of null models (see above). In this analysis of predictor ROI weights, to compare predictor weights within and across models, we normalized the predictor coefficients by taking their absolute value and dividing by the sum of absolute weights onto the modeled ROI. The weights were therefore expressed in % of the total weight.

### Controlling for correlation between head motion and age

Previous studies show correlation between head motion and age [21], which could have residual effects even after head-motion correction was applied [52,53]. Hence, when we examined correlations between subject age and prediction error, multiregional prediction gain, or predictor weight, we controlled for the correlations between head motion and age, by calculating the partial correlation (in Matlab) between the measure of interest and subject age while controlling for correlations between age and head motion estimated by the motion correction procedure. We used the estimated mean displacement between consecutive fMRI volumes scanned as an estimate for head motion [52].

### Tests of significance

All significance tests reported in this study for the difference in measures between models were determined pair-wise, using t-test in Matlab. When we conducted multiple comparisons, we corrected the *p* value using Bonferroni correction (multiplying the *p* value by the number of comparisons). Correlation between variables and its significance were determined using Pearson correlation coefficient in Matlab.

### Temporal signal to noise ratio (tSNR)

It is not trivial to estimate SNR in resting-state fMRI, and the noise also differs spatially. For the purpose of this study we used the temporal SNR [54] in each ROI, quantified by the average magnitude of % signal change divided by the variance of the time series, or:

tSNR=〈|x|〉/Var(x) (4)

Where *x* is the time series of the ROI (in % signal change from average).
